# Evaluation of clinical interventions made by pharmacists in chemotherapy preparation

**DOI:** 10.2478/v10019-010-0040-x

**Published:** 2010-09-22

**Authors:** Lea Knez, Raisa Laaksonen, Catherine Duggan

**Affiliations:** 1 Academic Department of Pharmacy, Barts and The London NHS Trust, Royal London Hospital, London, UK; 2 Pharmacy Department, University Clinic of Respiratory and Allergic Diseases Golnik, Golnik, Slovenia; 3 Pharmacy Practice Group, Department of Pharmacy & Pharmacology, University of Bath, Bath, UK; 4 Clinical Pharmacy – Development and Evaluation for East & South East England Specialist Services, NHS, UK; 5 The School of Pharmacy, London, UK

**Keywords:** pharmacy, cancer, chemotherapy, drug compounding, medication errors

## Abstract

**Background:**

Cancer drugs are high risk drugs and medication errors in their prescribing, preparation and administration have serious consequences, including death. The importance of a multidisciplinary approach and the benefits of pharmacists’ contribution to cancer treatment to minimise risk have been established. However, the impact of services provided by pharmacists to cancer patient care is poorly studied. This study explored the clinical interventions made by pharmacists in dispensing of chemotherapy doses, and evaluated pharmacists’ contribution to patient care.

**Methods:**

Pharmacists at the Chemotherapy Preparation Unit at a tertiary cancer centre in London were shadowed by two research pharmacists during the clinical screening of chemotherapy prescriptions and release of prepared drugs. An expert panel of pharmacy staff rated the clinical significance of the recorded interventions.

**Results:**

Twenty-one pharmacists’ interventions were recorded during the screening or releasing of 130 prescriptions or drugs. “Drug and therapy” (38%), “clerical” (22%) and “dose, frequency and duration” (19%) related problems most often required an intervention, identifying areas in chemotherapy prescribing that need improvement. The proposed recommendations were implemented in 86% of the cases. Many recorded interventions (48%) were ranked to have had a “very significant” influence on patient care.

**Conclusion:**

Clinical interventions made by pharmacists had a significant impact on patient care. The integration of pharmacists’ technical and clinical roles into dispensing of chemotherapy doses is required for providing high-quality cancer services.

## Introduction

Cancer drugs, involved in 15.4% of reported fatal cases, are second only to drugs acting on the central nervous system in medication error associated mortalities.^1^ High toxicity of cancer drugs is not problematic only when these medications are used inappropriately, but life-threatening side effects may occur also during regular treatment - their use requires clinical expertise. 2 Thus, cancer drugs are defined as high risk drugs; the prescribing, preparation and administration of which require special regulation and the collaboration of different healthcare professionals.^3^,^4^ Pharmacists have a central role in guaranteeing the safe, effective and economic use of cancer drugs.^3^–^8^

The dispensing of cancer drugs in designated centralized chemotherapy preparation pharmacy units has been extensively studied to improve its quality and minimise personnel exposure to these drugs.^4^–^7^ As a result, the technical roles, responsibilities and duties of pharmacists in the dispensing of chemotherapy doses are well defined.^5^–^7^ However, the same cannot be said of the clinical role of pharmacists. Although this role has been described to some extent in the Competency Framework for Specialist Oncology Pharmacists of the British Oncology Pharmacists Association and in the German Quality Standard for the Pharmacy Oncology Service as well as emphasised in numerous studies, the clinical services provided by pharmacists in chemotherapy dose dispensing are not standardised across Europe.^7^–^11^ This may lead to substantial variation in the quality of patient care.

As a consequence of being poorly defined, pharmacists’ clinical services in chemotherapy dose dispensing are also poorly studied. The implementation and benefits of these services have been described in several reports;^4^,^9^–^12^ however, pharmacists’ contribution to patient care is rarely assessed. Only a recent report of the British National Confidential Enquiry into Patient Outcome and Death (NCEPOD) provided evidence on the extent of pharmacists’ contribution to patient outcomes.^11^ The report analysed the treatment of cancer patients who had died within the first 30 days after receiving systemic chemotherapy. Finding that only half of chemotherapy prescriptions of these patients had been checked by a pharmacist, the NCEPOD highlighted this service as one of the measures required to reduce the risk of death after receiving systemic chemotherapy. The evaluation of current practices and the evaluation of the impact of changes in routine practices on patient care are important in all fields of oncology.^13^

This study aimed to explore clinical interventions made by pharmacists in dispensing chemotherapy doses, and to evaluate their significance for patient care.

## Methods

### Study design and sample

The study was designed as a prospective, descriptive, cross-sectional study of interventions made by pharmacists in dispensing of chemotherapy doses. The study complements another study that focused on exploring interventions made by pharmacists when providing routine clinical pharmacy services on cancer wards.^14^ The study was conducted at the Chemotherapy Preparation Unit (CPU) at St. Bartholomew’s Hospital, London, UK, a tertiary cancer centre. Ethics approval was not required as the study was part of the Trust’s service development. However, the study protocol was reviewed by two independent researchers at the School of Pharmacy, University of London, to assess any ethical issues.

**FIGURE 1.**
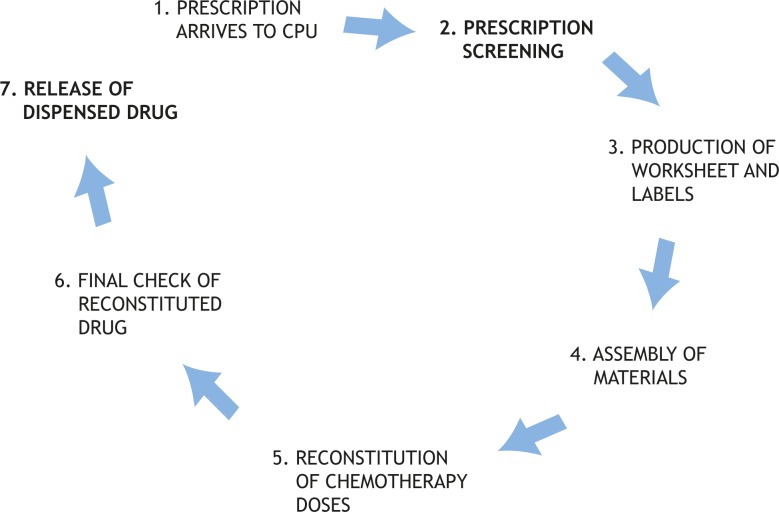
Stages in dispensing of cancer drugs in the Chemotherapy Preparation Unit (CPU). The study observed pharmacists during the stages of prescription screening and release of dispensed drugs; stages where only technical interventions were expected or where pharmacy technician and assistants were participating were not covered in the study.

The study evaluated interventions made by pharmacists during their daily routine practice in chemotherapy dose dispensing in a hospital setting (Figure 1). The study focused on the two stages in this process that can be performed only by pharmacists: screening of prescriptions; and release of dispensed drugs.

During prescription screening, pharmacists checked the correctness of clerical data, dose calculation, dose adjustment in altered essential pre-treatment investigation data, time and mode of administration and prescription of supportive therapy for expected toxicities. Before the release of a drug, pharmacists had to verify the correctness of the overall procedure and the quality of the dispensed drug.^5^ An intervention, such as adjusting a chemotherapy dose or adding supportive therapy, may be required at any point of the described processes. Both stages were observed at different times and independently of each other. Four clinical oncology pharmacists and one advanced clinical oncology pharmacist were working at the CPU at the time of the study.

### Developing the data collection form and collecting data

A literature review on potential medication errors and pharmacists’ clinical interventions^12^–^19^, observations of pharmacists’ work at the CPU, discussions with pharmacy practitioners and academic supervisors served as the basis for the development of a data collection form for recording and classifying the interventions. Interventions may be required due to various problems. Depending on the identified problem, an intervention could be classified to be required due to “a clerical problem”, for example, missing patient or administrative data, “a drug and therapy problem”, for example, omission or commission of drugs, or presence of a contraindication for the prescribed drug, “a dose, frequency and duration problem”, for example, inappropriate dose calculation or need for dose adjustment, wrong time or duration of the chemotherapy or “an administration and formulation problem”, for example, formulation or administration discrepancies with agreed treatment protocols.

The data collection was carefully planned. While the observation times were randomly selected, they allowed observations during six morning and three afternoon shifts on five different week days, taking into account potential variation in workload. The data were collected by two research pharmacists, who independently observed, without interfering, the clinical pharmacists’ work; this method has been found to be superior to self-reporting by healthcare professionals in medication error research.^20^

### Rating the significance of interventions

An expert panel of four members of pharmacy staff (the head of preparation services, two clinical oncology pharmacists and a specialist in drug manufacture and drug stability) was first asked to individually rate the interventions’ significance to patient care (patient safety) using a five-point Likert scale (Table 1).^20^ The clinical significance of the recorded interventions was then determined using a modified nominal group consensus method^20^, each panellist’s opinions were presented and discussed until the panel reached a consensus. To further validate the panel’s decisions, a consultant in medical oncology independently ranked the clinical significance of a sample of 13/21 (60%) interventions that were selected using a list of randomly generated numbers (Table 1).

### Data handling and statistical analysis

Confidentiality of patients and pharmacists was observed in handling the collected data and no names were recorded. The data on the recorded interventions were coded and entered onto an SPSS (version 14) database. Data are presented as frequencies and proportions; median values and ranges are presented where possible. Associations and differences between variables were explored using non-parametric tests: Chi square, Mann-Whitney U and Kruskal-Wallis H test, as appropriate.^21^

## Results

At the time of the study, the CPU received an average of 230 chemotherapy orders daily. Five pharmacists were observed during the screening of 85 prescriptions and the release of 45 drugs (Table 2). Overall, 21 interventions, which concerned 29 drugs prescribed for 18 patients, were recorded: 19 during prescription screening (19/85; 22%) and two during drug release (2/45; 4%).

### Patient characteristics

Patients, whose treatment required an intervention, had a median age of 49 years, ranging from 24 to 75, and most were female (15/18; 83%) (Table 3). More patients were treated for solid tumours (12/18; 67%) than for haematological malignancies. Patients were treated with standard chemotherapy (14/18; 78%) or received clinical trial treatment (4/18; 22%) and two patients received concomitant treatment with radiotherapy (2/18; 11%).

### Intervention characteristics

The identified problems were often related to “drug and therapy” (8/21; 38%), followed by “clerical” (7/21; 33%) and “dose, frequency and duration” (4/21; 19%) issues whereas “administration and formulation” problems (2/21; 10%) required an intervention of a clinical nature less often (Table 2). Some interventions (6/21; 28%) were required due to altered pre-treatment investigation data, for example, changes in drug doses were required because of out of range blood test results, or deteriorating renal or liver function.

Certain problem types, for example, altered investigation results, were often found to require similar interventions to rectify the problem, resulting in pharmacists making similar recommendations, for example, proposing a dose modification (Table 1). The identified problem and proposed recommendation were discussed, if needed, with the responsible clinician (13/21; 62%) or nurse (2/21; 10%). Most interventions (18/21; 86%) were implemented as recommended (Table 2). One was implemented with an amendment – instead of reducing the dose of carboplatin due to altered renal function, the clinician decided to postpone the chemotherapy cycle. In two cases the interventions were not accepted. In the first case, postponing of the chemotherapy treatment of a patient with grade three neutropenia had been recommended. A clinician argued that despite the chemotherapy having been previously postponed and the dose reduced the neutropenia had been persistent; thus, the patient should be treated. In the second case, while halving the dose of paclitaxel had been recommended due to altered hepatic function, a clinician offered no reason for not reducing the dose.

Most interventions concerned cancer drugs (27/29; 93%) than supportive therapy drugs. Since all cancer drugs are renowned as high risk drugs, pharmacists’ interventions prevented serious consequences of errors in their use. Some interventions did not involve any drug (5/21; 24%) (Table 2).

### Significance of the recorded interventions for patient care

The expert panel rated the interventions made by pharmacists: three had “significant” (14%); 10 “very significant” (48%); and one “potentially life saving” (5%) impact on patient care, preventing detrimental effects on the patients (Table 1). Moreover, the consultant in medical oncology independently ranked all of the 13 randomly selected interventions to be at least “significant” for patient care and gave exactly the same rating as the expert panel in six of 13 cases (Table 1). The consultant considered two interventions rated by the expert panel as having “minor significance” to patient care to be “potentially life saving”; these were the only two interventions where the rating differed by more than one category. The significance of recorded interventions was not associated with any patient characteristic or drug involved; no patient or drug subgroup was identified to be at greater risk of potential medication errors that would require greater vigilance.

Pharmacists were more likely to independently solve problems of minor significance, whereas they worked with clinicians and nurses to implement interventions of higher significance (Kruskal – Wallis, X^2^=10.686, df=2, p=0.005). Drug related problems related to “drug and therapy” and “dose, frequency and duration” were more likely to require interventions of higher significance than those related to “clerical” and “administration and formulation” issues (Kruskal – Wallis, X^2^=8.003, df=3, p=0.046).

Due to resource restraints it was not possible to observe all interventions made by pharmacists in one week. However, if the data are extrapolated on the weekly average of 230 chemotherapy prescriptions, pharmacists are expected to make approximately 50 interventions during prescription screening and ten during the release of dispensed drugs. Based on this estimation and the significance of the observed interventions, three drug related problems with potential fatal consequences may be prevented every week.

## Discussion

This study provided evidence of the contribution of the clinical pharmacists in the dispensing of chemotherapy doses to the safety of patients.

### Strengths and limitations

Studies of self-recorded clinical interventions may underestimate the frequency of required interventions^20^; in this study data were collected by independent researchers, providing a more complete picture of the interventions. Moreover, the expert panel reached consensus on the significance of the recorded interventions. However, the study presents some limitations.

This cross-sectional study was limited to nine visits in a centralized CPU at one hospital. The results may not be representative, but the study aimed at providing insight into pharmacists’ clinical interventions in dispensing of chemotherapy doses. The low number of visits was accepted as a limitation in exchange to having the data collected by independent observers. While the impact of pharmacists’ intervention on patient care was determined by an expert panel of pharmacy staff, one medical consultant separately evaluated the interventions, mainly confirming the panel’s decisions. Furthermore, the high level of significance assigned to the interventions by the consultant and the high proportion of implemented interventions suggest a high level of agreement and further confirm the need for similar services.

### Findings

The number of recorded interventions and their significance show that pharmacists contribute to patient care, which confirms the importance of their role in managing the risks associated with cancer drugs. Pharmacists’ interventions on prescribed chemotherapy observed on the wards and good and accurate risk management procedures in the dispensing of chemotherapy doses, such as the use of pre-written chemotherapy prescriptions, may have lowered the number of interventions needed as confirmed in the literature.^4^,^9^–^11^,^14^,^22^

The rate of interventions in the present study, 22% during prescription screening and 4% during drug release, was higher than the rate of medication errors identified in chemotherapy prescription orders reported in the literature.^9^,^11^ Markert *et al.* reported a chemotherapy error in 17.1% of received chemotherapy orders^9^, while Slama *et al.* reported a prescribing error in 12% of the received chemotherapy orders.^11^ However, the majority of errors in the described studies, 50.9% and 74%, respectively, were related to problem categories not included herein: missing a patient’s informed consent; and physicochemical incompatibilities. The lower proportion of recorded interventions in the literature may be attributed to differences in the duties of pharmacists in the dispensing processes in different countries or to discrepancies in the methodology of the studies.

The problems identified in the recorded interventions indicate areas in the chemotherapy prescribing practice that need improvement: writing of a chemotherapy order; and adjusting the dose according to blood and biochemistry data. Pharmacists were often observed to recommend changes to chemotherapy prescriptions that contained incorrect information; a problem that has been reported in the literature.^9^,^11^,^23^ Correct patient details on weight and height are of utmost importance for the correct calculation of the chemotherapy dose. Computerised chemotherapy order forms have been shown to diminish the number of errors^4^,^5^,^9^,^10^,^24^; while the possibilities for the implementation of computerised prescribing should be investigated, the limitations and problems of similar systems should be acknowledged.^25^,^26^

The individual dosing of cancer drugs and their important toxicity profile require constant monitoring of the health status of the patient. In this study, dose adjustment due to altered blood count, renal or hepatic function test results was the most common intervention, preventing the occurrence of potential adverse events with detrimental effects on the patient. This confirms the importance and need of pharmacists’ clinical knowledge in dispensing of chemotherapy doses. Moreover, the two interventions observed during the release of drugs also recommended a dose adjustment due to a change in patients’ renal or hepatic function. In both cases, the essential pre-treatment investigation data had not been available at the time of prescription screening, thus, to avoid a delay in dispensing the chemotherapy dose, the order was forwarded to the preparation room without this check, and the investigation data were available and checked only before the release of the drug, when the need for dose adjustment was identified. While most interventions (90%) have occurred before the release of drugs, such practice may result in the disposal of a dispensed drug and their re-dispensing; thus, the timely availability of essential pre-treatment investigation data may not have only safety, but also substantial economic implications. The literature shows that checking of chemotherapy doses with essential pre-treatment investigation has not been clearly stated as obligatory in many settings.^7^,^9^,^11^,^23^ However, the results of the present study imply that this service should be integrated into the dispensing of chemotherapy doses.

Pharmacists made highly significant interventions, showing the value of their contribution to cancer services. No patient characteristic or drug group was identified to require special interventions, perhaps due to low numbers of observations. The harm of medication errors in chemotherapy prescribing requires maximal risk control mechanisms *per se*, regardless of the treated patient or used drug. When dealing with problems of greater significance such as a contraindication for the prescribed treatment or a need for dose adjustment, pharmacists consulted nurses and clinicians. Clinicians agreed with most of the proposed interventions, confirming their importance also from a medical perspective. Good and well established collaboration between all healthcare professionals should be routine to prevent drug related problems from occurring in chemotherapy treatment.

The benefits of good collaboration are not restricted to chemotherapy dose dispensing. In fact, the collaboration between pharmacists and clinicians on the wards together with the tight regulation of the dispensing of chemotherapy doses may have solved problems of mainly minor significance^14^, before they reached the CPU. This, in addition to the fact that most interventions involved cancer drugs that are by definition high risk drugs, may have contributed to the high clinical significance of the recorded interventions. The integration of the work of pharmacists on the cancer wards and at the CPU is a great advantage for patient care.

To our knowledge, this study is the only study evaluating the impact of pharmacists’ clinical interventions in dispensing of chemotherapy doses on patient care. Studies describing pharmacists’ services in dispensing of chemotherapy doses either did not evaluate their impact on patient care or the contribution to patient care was done *a priori*, according to the detected medical error.^4^,^9^,^11^,^23^ However, the need for pharmacists’ contribution in high-quality services, shown in this study, coincides with other studies of pharmacists’ interventions in other ward settings.^14^–^19^,^22^ Clinical services, provided by pharmacists, were shown to be important in the treatment of cancer patients, who are exposed to complex treatment with high-risk drugs.

## Conclusions

This study showed the importance of the integration of pharmacists’ clinical and technical knowledge in the dispensing of chemotherapy doses to provide high quality cancer services. Pharmacists’ clinical activities in the dispensing of cancer drugs were shown to be essential for improving patient care and preventing major toxicity, and should be defined as a standard of care in guidelines, regulating the dispensing of chemotherapy doses of cancer drugs.

## Figures and Tables

**TABLE 1.** Description of the recorded interventions and their clinical significance

**SIGNIFICANCE FOR PATIENT CARE**		**INTERVENTION**
**Expert panel**	**Medical consultant**	
**Potentially life saving**	Potentially life saving	Trastuzumab is ordered for a patient, who has experienced a serious adverse drug event during previous administration.

**Very significant**	Potentially life saving	Chemotherapy is ordered 7 days ahead of protocol.
	Very significant	Impaired hepatic function requires dose modification of paclitaxel.
	Very significant	Etoposide dose was miscalculated when switching from oral to intravenous route of administration.
	Very significant	Impaired renal function requires dose modification of cisplatin.
	Significant	Impaired renal function requires dose modification of carboplatin.
	Significant	Chemotherapy is ordered as 6th cycle whereas it was patient’s 4th cycle.
	Significant	Chemotherapy order is not signed by the medical practitioner.
	NA*	Impaired renal function requires dose modification of fludarabin.
	NA	Grade 3 neutropenia require chemotherapy to be postponed.
	NA	Full dose of irinotecan is ordered although patient received modified doses in previous cycles.

**Significant**	Very significant	Two chemotherapy orders with different information on body surface area are received for the same patient.
	Significant	Cancer drug that is per protocol given every week interval is ordered every fortnight.
	Significant	The chemotherapy order does not include the required antiemetic therapy.

**Minor significance**	Potentially life saving	The name of the patient on chemotherapy order is illegible.
	Potentially life saving	Cancer drugs for iv and it administration are prescribed on same chemotherapy order.
	NA	The order for the last chemotherapy is not recorded in the CPU† patient file.
	NA	Incorrect information of a patient’s height.
	NA	Wrong calculation of the body surface area.
	NA	Grade 3 neutropenia require chemotherapy to be postponed.
	NA	Etoposide is instable in the ordered infusion volume.

* NA (not applicable) the medical consultant did not assess the clinical significance of interventions for patient care

† CPU stands for Chemotherapy Preparation Unit

**TABLE 2.** Characteristics of recorded interventions

**Parameter**	**Category**	**n**	**%**
**Number of recorded interventions**	Number of observations	130	
	Number of interventions	21	

BNF* group of drug involved	Malignant diseases & immunosupression	27 / 29†	93 %
	• Antimetabolites	6 / 29†	21 %
	• Anthracyclines & other cytotoxic antibiotics	5 / 29†	17 %
	• Vinca alkaloids & etoposide	4 / 29†	14 %
	• Other antineoplastic drugs: Taxanes	4 / 29†	14 %
	• Other antineoplastic drugs	8 / 29†	28 %
	Drugs outside malignant disease & immunosupression group	2 / 29†	7 %

**Identified drug related problem**	Drug and therapy	8 / 21	38 %
	Clerical	7 / 21	33 %
	Dose, frequency and duration	4 / 21	19 %
	Administration and formulation	2 / 21	10 %

**Contacted healthcare professional**	Clinician	13 / 21	62 %
	Nurse	2 / 21	10 %
	None	6 / 21	29 %

**Implementation of the intervention**	Implemented	18 / 21	86 %
	Implemented, with amendments	1 / 21	5 %
	Not implemented	2 / 21	10 %

* BNF stands for British National Formulary

† one intervention could involve more than one drug

**TABLE 3.** Characteristics of patients

**Characteristic**		**n**	**%**
**Sex**	Female	15 / 18	83 %
	Male	3 / 18	17 %
	
**Diagnosis**	Solid tumours	12 / 18	67 %
	• Lung cancer	4 /18	22 %
	• Breast cancer	3 / 18	17 %
	• Other solid tumours	5 / 18	28 %
	Haematological malignancies	6 / 18	33 %
